# Inhibition of nm23-H1 gene expression in chronic myelogenous leukemia cells

**DOI:** 10.3892/ol.2013.1500

**Published:** 2013-07-29

**Authors:** ZHENSHENG DAI, WEIZHONG XIAO, YUELING JIN

**Affiliations:** 1 Department of Hematology, Shanghai Pudong Hospital, Affiliated to Fudan University, Shanghai 201399, P.R. China; 2 Department of Neurology, Shanghai Pudong Hospital, Affiliated to Fudan University, Shanghai 201399, P.R. China; 3 Department of Pathology, Shanghai Health Vocational Technical College, Xuhui, Shanghai 200237, P.R. China

**Keywords:** nm23-H1, chronic myelogenous leukemia, RNAi, migration

## Abstract

For solid tumors of a malignant origin, the expression of the nm23-H1 gene is a positive prognostic factor. However, for chronic myeloid leukemia (CML), the prognostic role of nm23-H1 gene expression is unknown. The present study investigated the impact of nm23-H1 gene expression on the proliferation and migration of the CML K562 cell line to elucidate the association between nm23-H1 gene expression and CML cell survival. An RNAi lipo-recombinant plasmid of the nm23-H1 gene (pGCsi-nm23-H1) was constructed and transfected into the K562 cells. RT-PCR and western blotting were used to detect nm23-H1 mRNA and protein expression, respectively. The anchorage-independent growth ability of the transfected cells was observed in soft agar culture and the ability of the K562 cells to migrate was determined using a Transwell assay. Following the successful construction and transfection of the pGCsi-nm23-H1 plasmid into the K562 cells, nm23-H1 mRNA and protein expression levels were significantly lower compared with the control group. The stably-transfected pGCsi-nm23-H1 K562 cells exhibited a markedly increased ability to form colonies and the number and sizes of the colonies were significantly increased compared with those of the control. *In vitro*, the cells migrated through a Matrigel-coated membrane during incubation for 20 h. The Transwell assay revealed that the quantitative number of pGCsi-nm23-H1 K562 cells that migrated into the lower compartment of the invasion chamber was markedly increased compared with the control. In conclusion, nm23-H1 gene expression may inhibit K562 cell proliferation and migration. nm23-H1 may be a cancer suppressor gene and play a significant role in inhibiting the survival of CML cells.

## Introduction

Our knowledge of the pathogenesis of leukemia, including an understanding of its molecular mechanisms, has progressed as numerous studies have been undertaken with regard to the various aspects of gene therapy. The prognosis of patients with leukemia is closely associated with the invasion and metastasis of the malignant cells. The nm23-H1 gene is a tumor metastasis suppressor gene. The effect of the gene on the prognosis and tumor metastasis have been described in studies of solid tumors, including those of gall bladder (1,2) liver (3,4) and gastric (5) cancer.

For malignant tumors of the blood system, the expression of the nm23-H1 gene is a poor prognostic factor (6,7). Magyarosy *et al*(8) studied nm23-H1 expression in acute lymphoblastic leukemia and revealed that the expression of nm23-H1 in low-differentiated cells was higher than that in relatively well-differentiated cells. Therefore, the nm23-H1 gene was considered to be a prognostic marker for a variety of cancers of the blood system. The K562 cell line originates from chronic myeloid leukemia (CML). Currently, studies on the nm23-H1 gene in CML are rare. Therefore, in the present study, the RNAi technique was used to inhibit nm23-H1 gene expression in the K562 cell line to investigate the affects of nm23-H1 gene expression on the proliferation and migration of the K562 cells and to further clarify its correlation with prognosis for the molecular targeted treatment of CML.

## Materials and methods

### Cell lines

K562 cells were obtained from the Shanghai Institute of Cell Biology, Chinese Academy of Sciences (Shanghai, China). The cells were cultured at 37°C in humidified 5% CO_2_ in RPMI-1640 medium (Sigma, St. Louis, MO, USA), supplemented with 10% fetal bovine serum and 100 units/ml penicillin and streptomycin.

### Short hairpin RNA (shRNA) preparation and plasmid construction

Two pairs of shRNA sequences were designed, one according to the nm23-H1 sequence in GenBank (D1734) and the other sequence with no homology to the human sequence, which was used as a control. Each pair contained a unique 19-nt double-stranded sequence that was separated by a loop of 9-nt sequences (ttcaagaga). The oligonucleotide sequences of siRNA contained a *Bam*HI and *Hin*dIII site. Subsequent to the purification and restriction digestion, the oligonucleotides were ligated into the pGCsi plasmid (GeneChem Inc., Shanghai, China) with the polymerase III U6 promoter. The nm23-H1 recombinant plasmid was confirmed by sequencing and named pGCsi-nm23-H1.

### RNA extraction and semi-quantitative RT-PCR

Total RNA extraction was performed using TRIzol reagent (Takara, Shiga, Japan). The reverse transcription reaction was performed using 2 μg total RNA with a first strand cDNA kit (Takara), according to the manufacturer's instructions. PCR was performed in a 25-μl reaction volume containing 2 μl cDNA template, 10X buffer, 0.15 mM dNTP, 0.1 mM of each primer and 0.5 U Ex Taq Hot Start Version (Takara). The primers and the amplification conditions that were used in the PCR are listed in Table I. The final products were identified in 1.7% agarose gel and stained with ethidium bromide.

### Transfection assay

To generate the nm23-H1 siRNA-transfected K562 cells, 3 μg plasmid DNA was transfected into 1×10^5^ cells in a 60-mm dish using lipofectamine 2000 (Invitrogen, Carlsbad, CA, USA), according to the manufacturer's instructions. The transfected cells were selected in a medium containing 400 μg/ml G418 (Geneticin; Invitrogen) and the stable nm23-H1 siRNA-transfected cells were named pGCsi-nm23-H1 K562 cells. The control K562 cells were transfected with liposome and named the liposome K562 cells.

### Western blotting

Each group of cells was washed twice with phosphate-buffered saline (PBS), lysed for 10 min in hot water and centrifuged at 20,000 × g for 10 min. Total proteins (10 μl) were separated by 5% sodium dodecyl sulfate polyacrylamide gel electrophoresis (SDS-PAGE) and transferred onto a polyvinylidene fluoride (PVDF) membrane. Subsequent to being immersed in 10 ml 5% skimmed milk in TBST solution for 1 h, the membrane was incubated with primary and secondary antibodies. Human monoclonal anti-nm23-H1 (1:300; BD Biosciences Pharmingen, San Diego, CA, USA) and β-actin (1:500; Invitrogen) antibodies were used as the primary antibodies. Bovine anti-mouse IgG (1:2500; Santa Cruz Biotechnologies, Santa Cruz, CA) was used as the secondary antibody. Finally, images of the results were captured with an enhanced chemiluminescence (ECL) substrate.

### MTT assay

For the cell proliferation assays, each group of cells was plated in triplicate in 96-well plates at a density of 1×10^4^ cells/well and grown for 1, 2, 3, 4, 5, 6 and 7 days, respectively. A total of 20 μl 5 mg/ml MTT was added. Following a 4-h incubation period, the number of metabolically active cells was quantified.

### Colony formation assay

The cells (1×10^3^) were seeded into 6-well plates with 2 ml culture medium. Subsequent to a two-week incubation period in RPMI-1640 medium supplemented with 10% fetal bovine serum at 37°C and 5% CO_2_, the colonies were washed twice with PBS, stained with Giemsa, counted, visualized microscopically and had their images captured.

### Transwell assay

For the migration assays, the pGCsil-nm23-H1 K562 and control liposome K562 cells were cultured in RPMI-1640 medium supplemented with 10% fetal bovine serum at 37°C and 5% CO_2_. When the cells had grown to 80% confluence, they were incubated for 24 h in medium without fetal bovine serum. The cell culture supernatants were collected and preserved at −20°C for further use as epidermal growth factor (EGF). The undersides of the Transwell chamber membranes (BD Biosciences Pharmingen) were coated with 250 μl Matrigel gels mixed with 250 μl RPMI-1640 medium. Each group contained 1×10^5^ cells and was seeded on the Transwell chamber. Following this, 800 μl NIH3T3 EGF that was prepared previously was added to the 6-well plates. Following 24 h, the Matrigel gel on the upper sides of the membranes was removed using cotton swabs. The Transwell chamber membranes were fixed in 95% ethanol for 15 min. The cells that had migrated to the undersides of the membranes were stained with hematoxylin and eosin (HE) and counted by microscopy (x200). The results were determined by averaging the cell counts in five fields.

### Statistics

The data were analyzed using the SPSS software program (v 11.0; SPSS, Inc., Chicago, IL, USA). Non-parametric tests were performed using independent samples. The mean values were compared by a one-way ANOVA. P<0.05 was considered to indicate a statistically significant difference.

## Results

### Transfection assay

**Figure 1:**
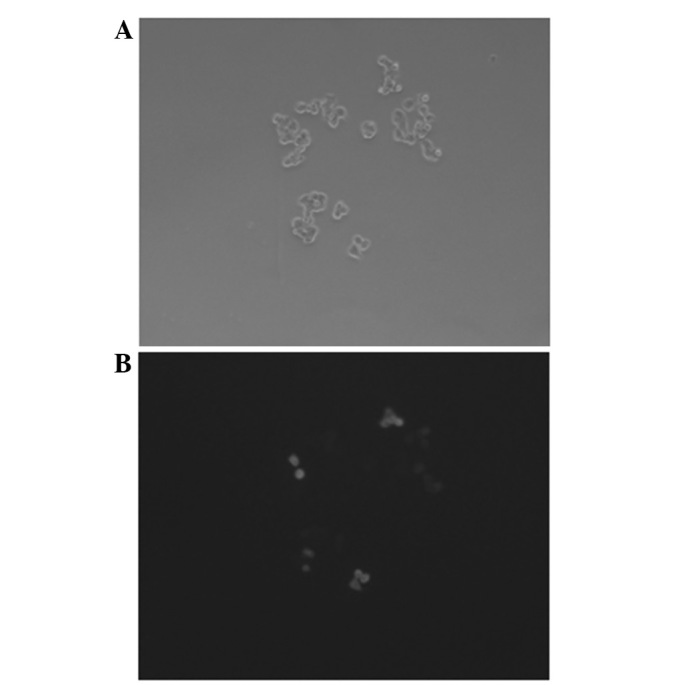
(A) Typical microscope image of pGCsi-nm23-H1 K562 cells. Slight changes in cell shape were observed. (B) Typical fluorescence microscope image of pGCsi-nm23-H1 562 cells. Green fluorescence is observed in the nucleus, which indicates that pGCsi-nm23-H1 has been transducted into the K562 cells successfully. (A and B) Magnification, ×40.

Following the transfection with the pGCsi-nm23-H1 plasmids, a change was observed in the morphology of the cells, and green fluorescence in the nucleus was visualized by fluorescence microscope. Following 48 h, the efficiency of the plasmid transfection was calculated. Plasmid transfection efficiency = number of fluorescent cells per high power field (HPF) / number of cells in the same field × 100 (3). The transfection efficiency of the pGCsi-nm23-H1-transfected K562 cells was ~40%. (Fig. 1)

### Inhibition of nm23-H1 gene expression by shRNA expression vectors

**Figure 2:**
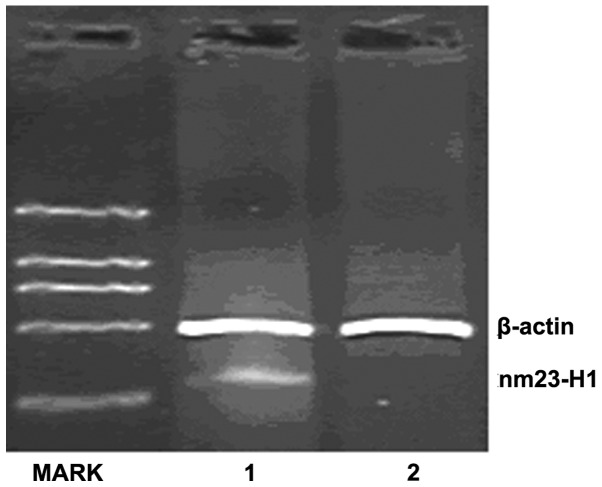
nm23-H1 mRNA expression. 1, liposome group; 2, pGCsi-nm23-H1 group. Inhibition of nm23-H1 expression in the K562 cell line. Semiquantitative PCR analysis was performed using total RNA extracted from the control and pGCsi-nm23-H1 K562 cells. β-actin was analyzed as a positive control. The expression of nm23-H1 mRNA was decreased in the pGCsi-nm23-H1 K562 cells. The sizes of nm23-H1 and β-actin were 252 bp and 457 bp, respectively.

**Figure 3:**
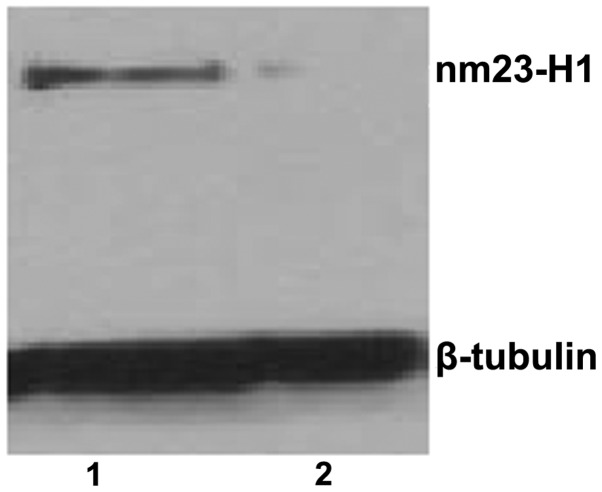
Western blot analysis of nm23-H1 expression. 1, liposome group; 2, pGCsi-nm23-H1 group. Bands of 40 kDa represent signals of β-tubulin and bands of 17 kDa represent signals of the nm23-H1 protein. Compared with the control group, a weaker expression of nm23-H1 was detected in the pGCsi-nm23-H1 K562 cells.

The knockdown efficiencies of nm23-H1-specific shRNA in the K562 cells were analyzed using semiquantitative PCR and western blotting. The relative nm23-H1 mRNA levels were normalized by internal control β-actin and the western blot assay for nm23-H1 protein expression was normalized by β-tubulin. Following transfection, the mRNA and protein expression levels of nm23-H1 were reduced in the pGCsi-nm23-H1 K562 cells. (Figs. 2 and 3)

### MTT assay

**Figure 4:**
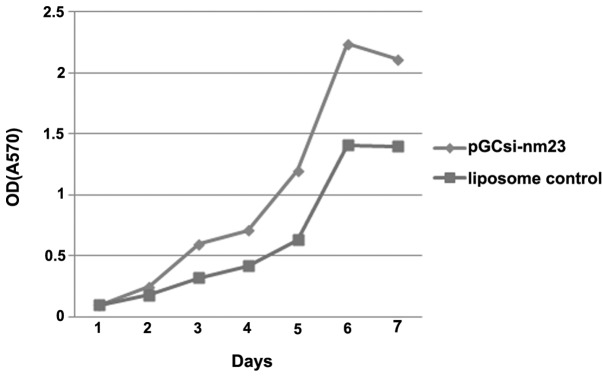
Cell proliferation was assessed by MTT assay. Cells were seeded in 96-well plates, grown for 1, 2, 3, 4, 5, 6 and 7 days and incubated with 20 μl 5 mg/ml MTT for 4 h. The quantification of the metabolic activity in the cells that were transfected with pGCsi-nm23-H1 was significantly higher than the cells of the liposome control groups, particularly after 4 days.

For the cell proliferation assays on the inhibition of nm23-H1 gene expression by the shRNA expression vectors, the number of metabolically active cells was quantified. The quantification of the metabolic activity of the cells that were transfected with pGCsil-nm23-H1 was significantly higher than that in the cells of the control groups, particularly following 4 days of transfection. (Fig. 4)

### nm23-H1-specific shRNAs induce cell forming colonies

**Figure 5:**
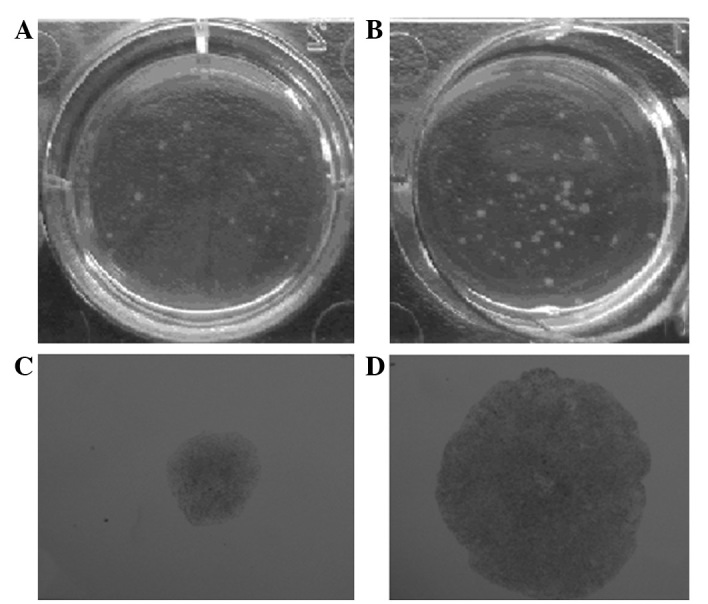
Inhibition of colony formation in K562 cells transfected with the nm23-H1 siRNA gene. Cells (1×10^3^) were seeded in 6-well plates and cultured for 2 weeks. The colonies were stained with Giemsa, counted and had their images captured. A significant increase in the colony number and volume was observed in the (B) nm23-H1 siRNA-transfected K562 cells compared with the (A) liposome K562 cells. The number of colonies that were formed were taken from three independent experiments. In the pGCsi-nm23-H1 group, the mean ± SD was 12.14±3.51, while in the liposome control group this value was 4.32±0.95, as visualized by microscopy (x40). The size of the colonies formed by the (D) pGCsi-nm23-H1 K562 cells were significantly increased compared with the (C) liposome control group. SD, standard deviation.

To evaluate the tumor suppression function of nm23-H1 in CML, the anchorage-independent growth abilities of the pGCsi-nm23-H1 K562 and liposome control K562 cells were compared in soft agar culture. The stably-transfected pGCsi-nm23-H1-siRNA K562 cells exhibited a dramatically increased ability to form colonies on soft agar. The number and sizes of the colonies that were formed by the pGCsi-nm23-H1 K562 cells were significantly increased compared with those that were formed by the liposome control group (Fig. 5).

### nm23-H1-specific shRNAs induce cell migration in vitro

**Figure 6:**
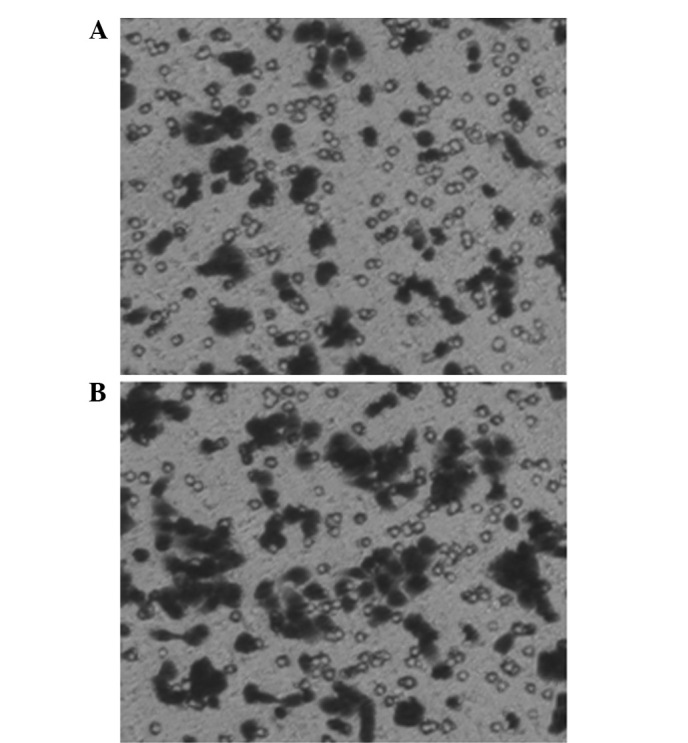
Detection of K562 invasiveness *in vitro* by the repression of nm23-H1 expression. The ability of pGCsi-nm23-H1 K562 cells to migrate across a Matrigel-coated membrane in an invasion chamber was evaluated. Following a 20-h incubation period at 37°C, the number of cells that had migrated across the membrane was determined. Invasive cells were stained, captured and counted under a microscope. (A and B) A representative result from three independent experiments with similar results. In the pGCsi-nm23-H1 group, the mean ± SD was 134.8±6.66, while in the liposome control group, this value was 54.4±3.42 (P<0.05). (A) Liposome group and (B) pGCsi-nm23-H1 group (x40). SD, standard deviation.

To analyze whether the nm23-H1 gene was involved in the migration of the K562 cells, the effect of the invasiveness of the pGCsi-nm23-H1 K562 cells was examined *in vitro*. The cells migrated through a Matrigel-coated membrane during a 20-h incubation period. The results revealed that the number of the pGCsi-nm23-H1 K562 cells that migrated into the lower compartment of the invasion chamber was markedly increased compared with the number of the liposome control K562 cells. Fig. 6 shows the mean ± standard deviation (SD) of three independent experiments (pGCsi-nm23-H1 group, 112.4±4.56; and control group, 68.4±2.40).

## Discussion

CML is a clonal myeloproliferative disorder that is characterized by the presence of the fusion oncogene, BCR-ABL. The constitutive expression of BCR-ABL leads to the unregulated production of mature myeloid cells in the bone marrow and their subsequent release into the blood (9). If untreated, CML will progress from a chronic to an accelerated phase over a number of years, prior to quickly proceeding to a terminal blast crisis phase, reminiscent of acute leukemia (10). The advent of tyrosine kinase inhibitors has led to an improved management of the disease. However, these drugs do not provide a cure as they are unable to eradicate the most primitive, quiescent fraction of CML stem cells (11).

The nm23-H1 gene is a metastatic suppressor that was identified in a melanoma cell line and is expressed in various tumors where their levels of expression are associated with a reduced or increased metastatic potential. nm23-H1 is one of >20 metastasis suppressor genes (MSGs) that have been confirmed *in vivo.* The gene is highly conserved from yeast to humans, implying a critical developmental function. Cell surface nm23-H1 has been previously observed in non-Hodgkin lymphoma (NHL) cells (12,13) and certain myeloid cell lines (14,15). Specific studies (13,14) have demonstrated that tumors with a reduced expression of the nm23 gene are more prone to metastasis. It has been also previously documented that the expression of nm23-H1 transcripts and, more so, the levels of nm23-H1 protein in serum, provide strong indicators of prognosis, with higher values being associated with poorer overall survival (13–15).

The present study revealed a strong association between nm23-H1 gene expression and K562 cell survival *in vitro*. The MTT assay demonstrated that the stably-transfected pGCsi-nm23-H1 K562 cells exhibited a markedly increased ability to form colonies on soft agar. The number and sizes of the colonies that were formed by the pGCsi-nm23-H1 K562 cells were significantly increased compared with those of the liposome control group. Furthermore, to test whether the nm23-H1 gene was involved in the migration of the K562 cells, the effect of the invasiveness of the pGCsi-nm23-H1 K562 cells was examined *in vitro*. The results revealed that the number of the pGCsi-nm23-H1 K562 cells that migrated into the lower compartment of the invasion chamber was markedly increased compared with the liposome control K562 cells. This suggests that the behavior of the nm23-H1 gene affects the biology of the CML cell lines, including growth, proliferation and invasiveness (16). These observations are consistent with other studies of solid tumors (17–19). However, the data from a study by Okabe-Kado *et al*(20) strongly indicated that the nm23-H1 gene may act as a tumor-derived survival factor in acute myeloid leukemia (AML). However, the study was unable to delineate between nm23-H1-binding AMLs and normal AMLs, in which the mechanism is likely to be active (20).

The experimental results from the present study suggest that the nm23-H1 gene is closely associated with the inhibition of metastasis. To assess the ultimate therapeutic potential of peptide vaccines derived from nm23, it will be necessary to determine, firstly, whether or not aberrant nm23-H1 expression is a widespread feature of CMLs and, secondly, whether the protein generates peptides that are able to act as functional antigens in HLA backgrounds other than HLA-A32. Given the widespread involvement of nm23 proteins in tumorigenesis, it will also be noteworthy to investigate the potential relevance of nm23-H2 as a therapeutic target in other cancers. The regulatory interdependence of nm23-H2 and c-myc provides a basis from which to design specific studies to elucidate the function of nm23 proteins in normal and leukemic cells, which may contribute to our understanding of the molecular mechanisms underlying the development and progression of CML (21). Future studies should investigate the association between nm23-H1 binding and responses to CML therapies, and aim to determine the nature of the nm23-H1 receptor in CML, which may provide a novel target for adjunctive therapies.

## Figures and Tables

**Table I:** List of primer sequences and amplification conditions used in the PCR.

Gene	Primer sequences (5′-3′)	PCR conditions	Product size (bp)
nm23-H1	5′-TTAATCAGATGGTCGGGGAT-3′ 5′-GATCTATGAATGACAGGAGG-3′	94°C, 30 sec; 56°C, 30 sec, 72°C, 30 sec; 32 cycles	186
β-actin	5′-CGTGGCCTTAGCTGTGCT-3′ 5′-TGTGCATAAAGTGTAAGTGTATAAGCA-3′	94°C, 30 sec; 54°C, 30 sec; 72°C, 30 sec; 32 cycles	457
